# Primary hepatic diffuse large B-cell lymphoma in a patient with polycystic liver disease: a case report and literature review

**DOI:** 10.3389/fonc.2026.1821947

**Published:** 2026-05-08

**Authors:** Jia-Sheng Ding, Min Zhang, Fangfang Zhou

**Affiliations:** 1 Department of Intensive Care Unit, Lishui Central Hospital, The Fifth Affiliated Hospital of Wenzhou Medical University, Lishui, Zhejiang, China; 2 Department of Pathology, Lishui Central Hospital, The Fifth Affiliated Hospital of Wenzhou Medical University, Lishui, Zhejiang, China; 3 Department of Ultrasound, Lishui Central Hospital, The Fifth Affiliated Hospital of Wenzhou Medical University, Lishui, Zhejiang, China

**Keywords:** case report, contrast-enhanced ultrasound, diffuse large B-cell lymphoma, PET/CT, polycystic liver disease, primary hepatic lymphoma

## Abstract

Primary hepatic lymphoma (PHL) is an extremely rare extranodal non-Hodgkin lymphoma confined to the liver, accounting for <0.016% of all non-Hodgkin lymphomas. Polycystic liver disease (PLD) is a benign genetic disorder characterized by multiple hepatic cysts. The coexistence of PHL in a patient with PLD has not been previously reported. A 68-year-old woman with a long-standing history of polycystic liver and kidney disease presented with progressive abdominal distension. Laboratory tests showed markedly elevated lactate dehydrogenase (947 IU/L) and soluble interleukin-2 receptor (599 U/mL), with normal tumor markers. Contrast-enhanced CT identified a mass in the right hepatic lobe, and ^18^F-FDG PET/CT revealed a large hypermetabolic lesion (SUVmax 22.5) without extrahepatic involvement. Contrast-enhanced ultrasound (CEUS) using Sonazoid demonstrated peripheral rim-like hyperenhancement in the arterial phase, rapid wash-out at 50 seconds, and absent enhancement in the Kupffer phase at 10 minutes post-injection, findings that guided targeted biopsy. Ultrasound-guided liver biopsy confirmed diffuse large B-cell lymphoma (DLBCL), non-germinal center B-cell (non-GCB) subtype, with immunohistochemistry showing CD20+, CD79a+, Bcl-6+, MUM1+, Bcl-2+ (80%), c-Myc+ (20%), Ki-67 (85%), and EBER negative. Bone marrow biopsy showed no involvement. The patient was treated with six cycles of R-CHOP chemotherapy. One year after treatment completion, restaging confirmed complete remission with no evidence of disease recurrence. To our knowledge, this is the first reported case of primary hepatic DLBCL occurring in a patient with polycystic liver disease. The diagnostic challenges posed by the polycystic background highlight the potential value of multimodal imaging, particularly contrast-enhanced ultrasound with Sonazoid, in characterizing lesions in this setting. While longer follow-up is needed, this case suggests that PHL should be considered in the differential diagnosis of new solid liver lesions in PLD patients, and that standard immunochemotherapy may achieve complete remission even in patients with underlying liver disease. Histopathological confirmation remains essential for diagnosis.

## Introduction

Primary hepatic lymphoma (PHL) is a rare extranodal non-Hodgkin lymphoma confined to the liver without involvement of lymph nodes, spleen, bone marrow, or other organs at the time of diagnosis (1, 2). PHL accounts for approximately 0.4% of extranodal lymphomas and 0.016% of all non-Hodgkin lymphomas (3). The most common histologic subtype is diffuse large B-cell lymphoma (DLBCL), accounting for 60-80% of cases (4, 5). PHL typically presents in middle-aged to older adults (median age 50–60 years) with a male predominance (6). Clinical manifestations are nonspecific and may include abdominal pain, fever, weight loss, hepatomegaly, and elevated lactate dehydrogenase (LDH) (7).

Polycystic liver disease (PLD) is a genetic disorder characterized by the progressive development of multiple hepatic cysts. It can occur as isolated PLD or in association with autosomal dominant polycystic kidney disease (ADPKD) (8). Patients with PLD are generally considered to have a benign prognosis, with most remaining asymptomatic and maintaining normal liver function (9). Malignancies arising in the setting of PLD are exceedingly rare, with only isolated reports of hepatocellular carcinoma (HCC) and cholangiocarcinoma (CCA) documented in the literature (10, 11). To confirm the novelty of this case, we performed a literature search in PubMed and Web of Science up to December 2024 using the following terms: (“primary hepatic lymphoma” OR “primary liver lymphoma”) AND (“polycystic liver disease” OR “polycystic liver” OR “PLD”). No language restrictions were applied. No previous cases of primary hepatic lymphoma arising in a patient with polycystic liver disease were identified.

## Case presentation

A 68-year-old woman with a known history of polycystic liver and kidney disease for over 10 years presented with progressive abdominal distension and dull pain in the right upper quadrant for one week, on a background of chronic abdominal distension. The patient reported fatigue and poor appetite but denied fever, night sweats, weight loss, nausea, vomiting, cough, or dyspnea.

Her medical history was unremarkable, with no history of viral hepatitis, HIV, or immunosuppressive therapy. Family history was non-contributory.

Physical examination revealed a significantly distended abdomen that was soft and non-tender, with no palpable masses or shifting dullness. The liver was markedly enlarged due to the underlying polycystic changes but was not tender. No peripheral lymphadenopathy or jaundice was observed.

Laboratory findings on admission were as follows: lactate dehydrogenase (LDH) 947 IU/L (reference range 110–220 IU/L), soluble interleukin-2 receptor (sIL-2R) 599 U/mL (reference range 122–496 U/mL), and aspartate aminotransferase 80 IU/L (reference range <35 IU/L). Alanine aminotransferase was within the normal range. Baseline renal function showed serum creatinine 0.8 mg/dL (reference range 0.5–1.1 mg/dL) and estimated glomerular filtration rate (eGFR) 81 mL/min/1.73m². Liver function tests, aside from mildly elevated AST (80 IU/L), were within normal limits including total bilirubin (0.7 mg/dL) and albumin (4.1 g/dL). Tumor markers including alpha-fetoprotein (AFP), carcinoembryonic antigen (CEA), cancer antigen 19-9 (CA19-9), and CA125 were within normal limits. Viral markers for hepatitis B, hepatitis C, and HIV were negative. Complete blood count and coagulation profile were normal.

Initial imaging began with contrast-enhanced CT, which revealed an irregular liver contour with multiple cystic lesions characteristic of polycystic disease. More importantly, a poorly defined hypodense mass measuring 7.3 × 6.2 cm was identified in the right hepatic lobe, demonstrating mild heterogeneous enhancement in the arterial phase with progressive filling in later phases (**Figure 1**). Diffuse peritoneal thickening and ascites were also noted. Given the contrast-enhanced CT findings of a solid mass with progressive enhancement and peritoneal changes, further characterization with functional imaging was pursued to assess metabolic activity and exclude extrahepatic disease, which is critical for differentiating primary from secondary hepatic lymphoma. Therefore, ^18^F-FDG PET/CT was performed next.

**Figure 1:**
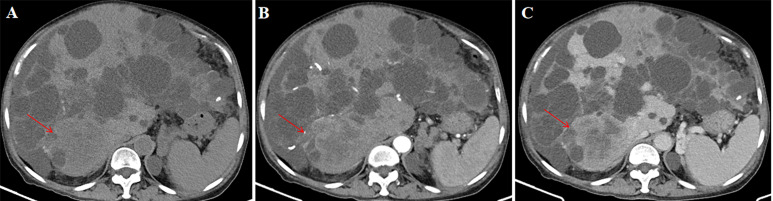
Contrast-enhanced CT findings of the hepatic lesion. **(A)** Axial non-contrast CT image shows a solid low-density mass (arrow) in the right hepatic lobe against a background of numerous cysts. **(B)** Arterial phase image demonstrates mild heterogeneous enhancement of the mass (arrow). **(C)** Delayed phase image shows progressive enhancement of the lesion (arrow).

This study confirmed a large hypermetabolic mass in the right hepatic lobe measuring 7.4 × 6.6 cm with intense FDG uptake (SUVmax 22.5) (**Figure 2**). The remainder of the liver parenchyma showed multiple cystic lesions with no FDG uptake, the largest measuring 9.3 cm in diameter, some with calcified walls. Both kidneys were enlarged with diffuse cystic lesions and no FDG uptake. Additional findings included peritoneal thickening without FDG uptake, ascites, dilated tortuous retroperitoneal vessels, bilateral small pleural effusions, and mild inflammatory changes in both lungs. Crucially, no abnormal FDG uptake was observed in lymph nodes, spleen, bone marrow, or other organs, suggesting that the disease was confined to the liver.

**Figure 2:**
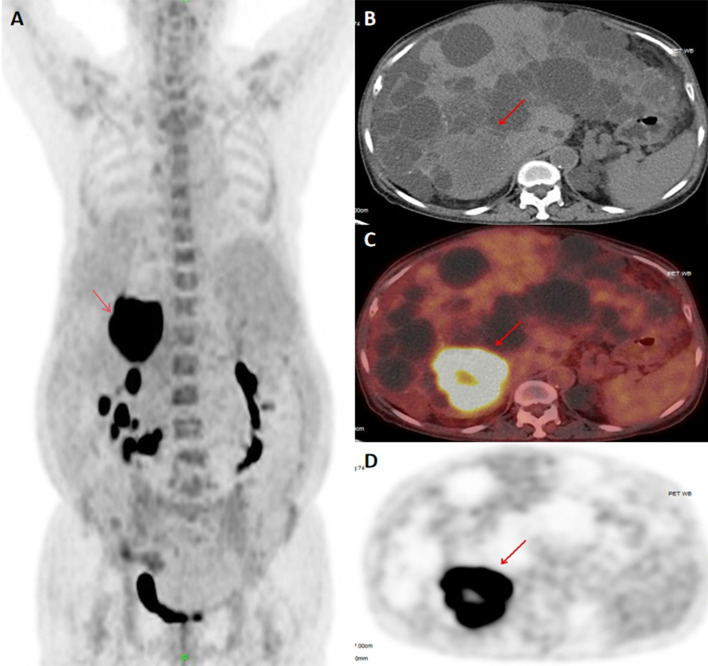
^18^F-FDG PET/CT findings. **(A)** Maximum intensity projection (MIP) PET image reveals intense FDG uptake in the right hepatic lobe (arrow) with no evidence of abnormal uptake elsewhere. **(B)** Axial CT image shows the corresponding soft tissue mass (arrow). **(C)** Fused PET/CT image demonstrates intense FDG uptake localized to the mass (arrow). **(D)** Axial PET image confirms the hypermetabolic nature of the lesion (arrow).

For tissue diagnosis and to guide biopsy, contrast-enhanced ultrasound using Sonazoid was performed. Gray-scale ultrasound revealed a hypoechoic nodule in the posterior right hepatic lobe measuring 7.1 × 7.0 cm. After intravenous injection of 0.5 mL Sonazoid (perfluorobutane microbubbles; GE Healthcare), which corresponds to the recommended clinical dose of 0.015 mL encapsulated gas per kilogram body weight, the lesion showed enhancement starting at 11 seconds. In the arterial phase, peripheral rim-like heterogeneous hyperenhancement was observed (**Figure 3A**). Wash-out began at 50 seconds in the portal phase (**Figure 3B**). The Kupffer phase image obtained 10 minutes post-injection revealed complete absence of contrast uptake within the lesion, indicating lack of phagocytic activity (**Figure 3C**). Ultrasound-guided biopsy was preferred over CT guidance because it allows real-time visualization of the lesion, avoids ionizing radiation, and, with contrast enhancement, enables precise targeting of viable tumor tissue while avoiding necrotic areas. The absence of Kupffer phase enhancement on Sonazoid-CEUS helped identify the most metabolically active peripheral portion of the mass for sampling. Ultrasound-guided liver biopsy was then performed, obtaining two core samples from the right hepatic mass using an 18G needle (**Figure 3D**). The specimens were sent for histopathological examination. Histopathological examination showed extensive necrosis and fibrotic tissue with scattered atypical degenerative cells (**Figure 4A**). Immunohistochemistry revealed the following profile: CD20+, CD79a+, Bcl-6+, MUM1+, Bcl-2+ (80%), c-Myc+ (20%), Ki-67 (85%) (**Figure 4B**); negative for CD3, CD5, CD10, CyclinD1, AE1/AE3, CK19, and INSM1. *In situ* hybridization for Epstein-Barr virus-encoded RNA (EBER) was negative. These findings were consistent with diffuse large B-cell lymphoma, and according to the Hans algorithm (CD10-, Bcl-6+, MUM1+), the tumor was classified as the non-germinal center B-cell (non-GCB) subtype.

**Figure 3:**
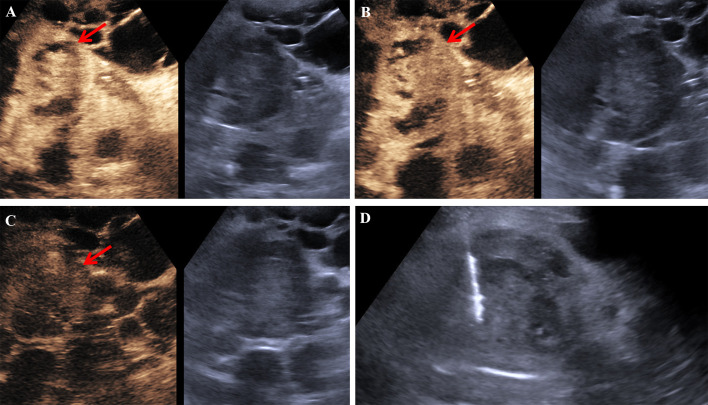
Contrast-enhanced ultrasound and ultrasound-guided biopsy of the hepatic lesion. **(A)** Arterial phase image obtained 11 seconds after Sonazoid injection shows peripheral rim-like heterogeneous hyperenhancement of the lesion, with enhancement occurring earlier than the surrounding liver parenchyma (arrow). **(B)** Portal phase image at 50 seconds demonstrates early wash-out with the lesion becoming hypoechoic relative to adjacent liver tissue (arrow). **(C)** Kupffer phase image at 10 minutes post-injection reveals complete absence of contrast uptake within the lesion, indicating lack of phagocytic activity (arrow). **(D)** Ultrasound-guided biopsy image shows the needle trajectory targeting the viable peripheral portion of the lesion as determined by prior contrast-enhanced imaging.

**Figure 4:**
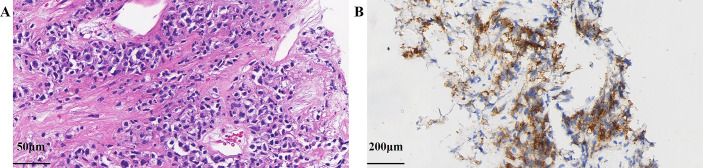
Histopathological and immunohistochemical findings of the liver biopsy. **(A)** Hematoxylin and eosin staining shows diffuse infiltration by large atypical lymphoid cells (original magnification ×400). **(B)** Immunohistochemical staining demonstrates strong positivity for CD20 (original magnification ×100).

Bone marrow biopsy from the posterior iliac crest showed normocellular marrow (50% cellularity) with trilineage hematopoiesis and no evidence of lymphoma infiltration. Reticulin fibrosis was absent.

Based on the absence of lymph node, splenic, bone marrow, or other extranodal involvement, a diagnosis of primary hepatic diffuse large B-cell lymphoma was established.

The patient was initiated on R-CHOP chemotherapy (every 21 days) with the following regimen: rituximab 600 mg IV on day 0, cyclophosphamide 1200 mg IV, vindesine 4 mg IV, liposomal doxorubicin 40 mg IV on day 1, and prednisone 100 mg orally on days 1–5. During the six cycles of R-CHOP, the patient experienced grade 1–2 neutropenia (nadir absolute neutrophil count 1.2 × 10^9^/L) without febrile neutropenia or documented infection. No dose reduction or treatment delay was required. Serial monitoring showed stable renal function (serum creatinine range 0.7–0.9 mg/dL) and no worsening of liver function. There were no grade 3 or 4 adverse events. The patient completed all six cycles without interruption.

One year after completing six cycles of R-CHOP chemotherapy, the patient underwent restaging evaluation. Contrast-enhanced CT showed complete resolution of the previously identified hepatic mass with no evidence of recurrent disease. Clinical examination revealed improvement in abdominal distension, and the patient had resumed her normal daily activities. She remained in good health with no signs of disease recurrence at one-year follow-up (**Table 1**).

**Table 1:** Laboratory findings at admission and one-year follow-up.

Parameter	Admission	Reference range	One-year follow-up
LDH (IU/L)	947	110–220	182
sIL-2R (U/mL)	599	122–496	412
AST (IU/L)	80	<35	41
ALT (IU/L)	32	<40	26
Serum creatinine (mg/dL)	0.8	0.5–1.1	0.9
eGFR (mL/min/1.73m²)	81	>60	70

## Discussion

Primary hepatic lymphoma (PHL) is a rare malignancy, representing less than 1% of extranodal lymphomas (1). Herein we describe the first case of primary hepatic diffuse large B-cell lymphoma arising in a patient with polycystic liver disease, a previously unreported coexistence. This case highlights the extreme rarity of PHL in general, the diagnostic challenges posed by the polycystic background, the utility of multimodal imaging in lesion characterization, and the excellent response to standard immunochemotherapy with durable remission. The diagnosis requires strict exclusion of lymphomatous involvement outside the liver at presentation (2, 12). In our patient, PET/CT and bone marrow biopsy confirmed disease confined to the liver, meeting the diagnostic criteria for PHL. The majority of PHL cases are of B-cell origin, with DLBCL being the most common subtype (4, 5, 13). Our patient’s immunohistochemical profile (CD20+, Bcl-6+, MUM1+, non-GCB subtype) is consistent with activated B-cell-like DLBCL, which carries a less favorable prognosis but remains chemosensitive (14). The high Ki-67 index of 85% indicates aggressive tumor biology. However, it is important to note that c-Myc expression was only 20% (with a positive threshold typically ≥40%), and Bcl-2 was 80%. Therefore, this case does not meet the criteria for a “double-expressor” lymphoma, which requires concurrent high expression of both c-Myc (≥40%) and Bcl-2 (≥50%). The absence of double-expressor status, together with the non-GCB subtype, is generally associated with retained chemosensitivity to R-CHOP-based regimens, a feature that may have contributed to the favorable clinical course observed in this patient.

PLD is a benign condition, and malignant transformation within cysts is exceptionally rare (10, 11). The relationship between PLD and PHL remains speculative. Polycystic liver disease is characterized by progressive cyst formation, chronic low-grade inflammation, and remodeling of the hepatic microenvironment. Some studies have suggested that chronic inflammation may promote lymphomagenesis through sustained antigenic stimulation and dysregulation of immune surveillance, but direct evidence linking cyst-induced inflammation to hepatic lymphoma is lacking. In our patient, the absence of hepatitis C, HIV, or other known risk factors for PHL raises the possibility of a coincidental association rather than a causative one. However, given the extreme rarity of both conditions, larger cohort studies would be needed to determine whether PLD confers any increased risk of hepatic lymphoma. At present, this case should be regarded as an unusual coexistence rather than evidence of a pathophysiologic link.

The diagnostic challenge in this case was compounded by the polycystic liver background, which can obscure solid lesions on conventional imaging. The sequential application of imaging modalities proved essential. Contrast-enhanced CT first raised suspicion by identifying a solid mass amidst the cystic background. PET/CT then confirmed the hypermetabolic nature of the lesion and, importantly, excluded extrahepatic involvement, supporting the diagnosis of primary rather than secondary hepatic lymphoma. Finally, contrast-enhanced ultrasound with Sonazoid provided critical real-time information about vascular perfusion patterns and Kupffer phase characteristics, confirming malignancy and guiding biopsy to viable tumor tissue while avoiding necrotic areas. Unlike PET-CT and contrast-enhanced CT, Sonazoid-CEUS delivers this real-time vascular and Kupffer phase data with higher precision, making it a more reliable tool for guiding targeted biopsy and avoiding necrotic tissue in PLD patients with complex hepatic backgrounds (15, 16). The absence of enhancement in the Kupffer phase, which reflects the lack of phagocytic activity in lymphoma tissue, was particularly valuable for confirming the malignant nature of the lesion (17). This logical progression from detection to characterization to tissue diagnosis exemplifies the value of multimodal imaging in complex cases.

The peritoneal thickening and ascites observed on CT and PET/CT showed no FDG uptake, arguing against malignant peritoneal involvement. These findings were therefore attributed to reactive changes secondary to the patient’s longstanding polycystic liver disease and associated portal hypertension, rather than lymphomatous spread.

The peripheral rim-like hyperenhancement with early wash-out observed on CEUS in our patient has been described in some PHL cases and may mimic intrahepatic cholangiocarcinoma (18). This pattern underscores the importance of tissue diagnosis even when imaging suggests a more common malignancy.

The differential diagnosis of a hypermetabolic liver mass in a patient with PLD includes cholangiocarcinoma, hepatocellular carcinoma, metastatic disease, and lymphoma. The absence of elevated tumor markers and the lack of a primary extrahepatic tumor argued against carcinoma. The elevated sIL-2R level, although nonspecific, raised suspicion for lymphoma (19). Histopathology with immunohistochemistry remains the gold standard for diagnosis.

Treatment of PHL follows guidelines for systemic DLBCL, with R-CHOP chemotherapy being the standard of care (20). Surgical resection is not routinely recommended due to the systemic nature of lymphoma. Given the patient’s underlying polycystic kidney disease, liposomal doxorubicin was selected over conventional doxorubicin to reduce potential renal stress while also minimizing cardiotoxicity. Our patient achieved complete response with durable remission at one year, as confirmed by comprehensive restaging including cross-sectional imaging, laboratory evaluation, and clinical assessment. This favorable outcome highlights the chemosensitivity of PHL even in patients with underlying liver disease.

The limitation of this case is that no subsequent ^18^F-FDG PET/CT examination was performed to confirm complete metabolic response, as complete metabolic response is considered the gold standard for evaluating the efficacy of diffuse large B-cell lymphoma (DLBCL).

## Conclusion

We present the first reported case of primary hepatic diffuse large B-cell lymphoma in a patient with polycystic liver disease. The polycystic background posed diagnostic challenges, which were addressed by a sequential multimodal imaging approach: contrast-enhanced CT for initial detection, ^18^F-FDG PET/CT to exclude extrahepatic involvement, and Sonazoid-enhanced ultrasound to guide targeted biopsy. Despite an aggressive immunohistochemical profile (non-GCB subtype, Ki-67 85%), the patient achieved complete remission after six cycles of R-CHOP and remained disease-free at one-year follow-up. Although PHL should be considered in the differential diagnosis of new solid hepatic lesions in PLD patients—particularly when tumor markers are normal—histopathological confirmation remains essential. Longer follow-up and additional cases are needed to further characterize this rare association.

## Data Availability

The original contributions presented in the study are included in the article/supplementary material. Further inquiries can be directed to the corresponding author.
